# Lithium induced chronic renal disease: A case report

**DOI:** 10.1016/j.radcr.2023.11.014

**Published:** 2023-11-29

**Authors:** Destiny Duvall, Tracy VanMeter

**Affiliations:** Department of Radiology, University of Louisville Hospital, 530 South Jackson St, CCB-C07, Louisville, KY, 40202 USA

**Keywords:** Lithium, Chronic renal disease, Cystic renal lesions, Bipolar disorder

## Abstract

We present a case of lithium-induced chronic renal disease in a 69-year-old female with past medical history of hypertension, and bipolar disorder, treated with long-term lithium-causing chronic renal disease.

## Introduction

The wide-spread use of lithium as a mood stabilizer began in the mid-19th century and continues to remain a front-line treatment for bipolar disorder today [1]. Epidemiologic reviews have suggested a lifetime prevalence of bipolar disorder of around 1% in the general population [2]. The acute renal toxicity of lithium has been well documented for decades but the link between chronic lithium use and the development of chronic renal disease (defined as eGFR <60 mL/min) progressing to end-stage renal disease has only relatively recently been documented. The risk of the development of renal failure after treatment with lithium has been shown to be age-dependent and increases with the duration of treatment with a hazard ratio of 2.5% (95% CI 1.6-4). This risk remains even after cessation of lithium therapy [3], [4], [5], [6]. Lithium toxicity occurs due to lithium accumulating in the collecting renal tubular cells, where lithium down-regulates vasopressin and can cause tubulointerstitial disease leading to the development of focal segmental glomerulosclerosis and microcyst formation [7]. Lithium-induced chronic renal disease has unique imaging characteristics both on ultrasound and magnetic resonance imaging demonstrating normal-sized kidneys with numerous uniform microcysts distributed both within the medulla and in the renal cortex [8].

## Case presentation

A 69-year-old woman with a history of bipolar disorder. She had been treated with long-term lithium which caused progressive renal disease and secondary hypertension. She initially presented to the outpatient nephrology office for evaluation and management of her known renal disease after recently moving to the area. Her past medical history was significant for grade 1 hypertension, treated with lisinopril and bipolar 1 disorder, currently treated with lamotrigine.

The patient reported that she had recently been transitioned to lamotrigine for her bipolar disorder but had previously been treated with lithium for 44 years. The decision to stop lithium was made by her prior psychiatrist after a progressive decline in her eGFR on routine lab work. Her bipolar disorder has remained in remission since the change in her mood stabilizer. The patient reported occasional polyuria and polydipsia.

Evaluation by nephrology reported that her eGFR continued to decline even after cessation of lithium therapy. Further evaluation was completed with updated bloodwork and point-of-care renal ultrasound in the office. Laboratory values were significant for eGFR>20, BUN 48, Creatine 2.4, and Calcium 10. The physical exam was significant for 2+ bilateral lower extremity edema.

**Fig. 1:**
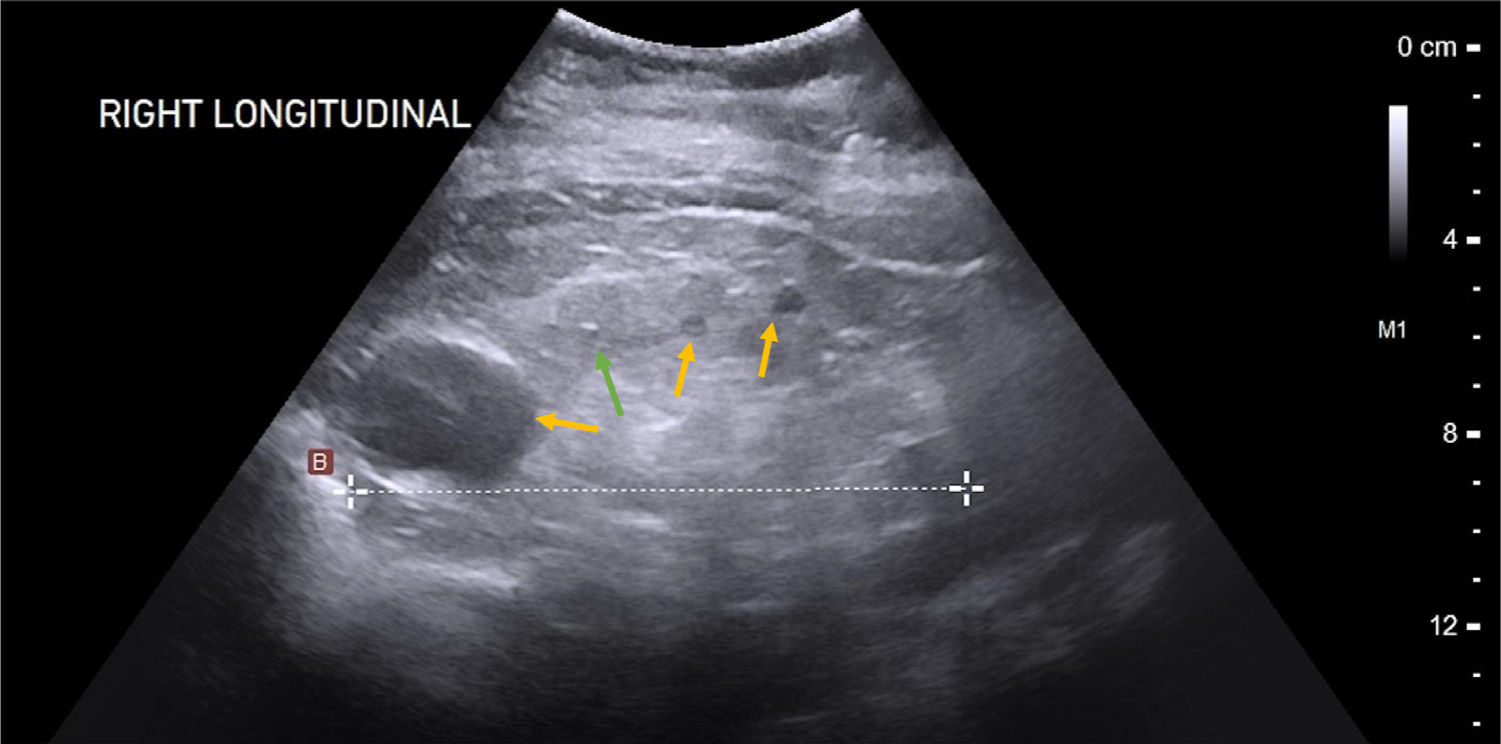
Point of care ultrasound performed in nephrology office of the right kidney. Multiple anechoic, well circumscribed cystic structures (orange arrows) and scattered echogenic foci (green arrow) are identified.

**Fig. 2:**
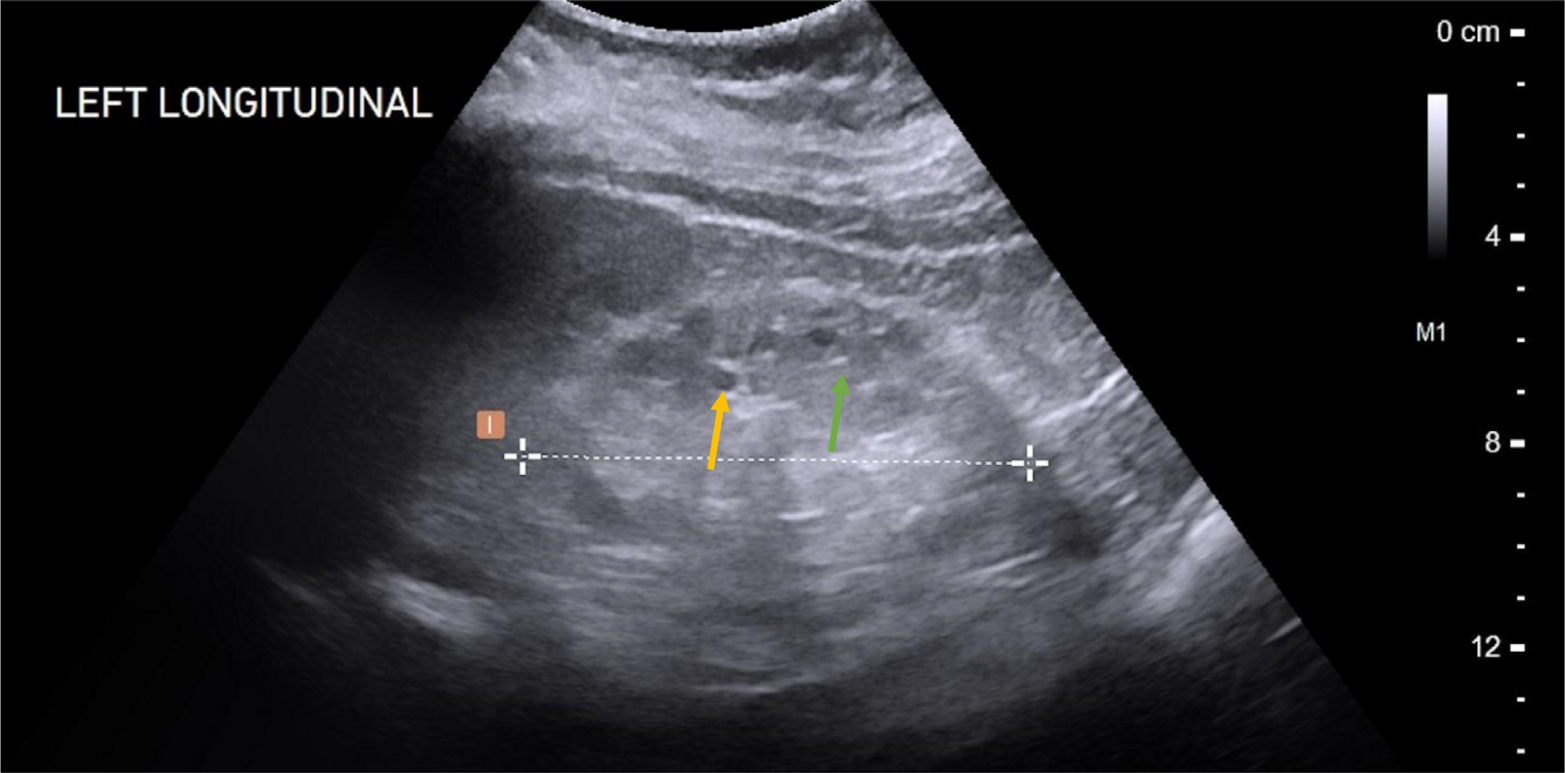
Point of care ultrasound performed in nephrology office of the left kidney. Multiple anechoic, well-circumscribed cystic structures and scattered echogenic foci are identified.

**Fig. 3:**
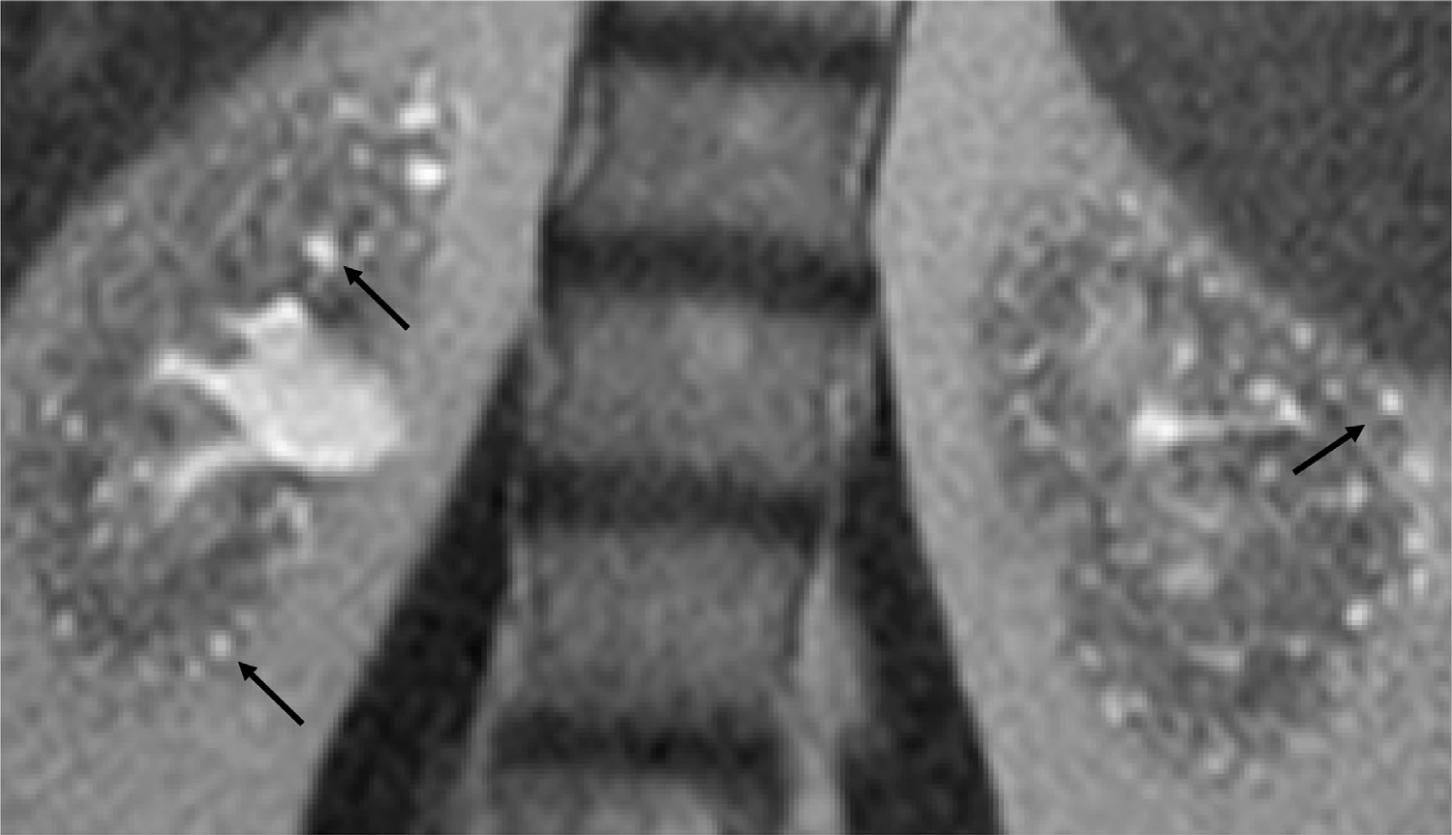
Select coronal T2 slice from MRI demonstrates normal-sized kidneys with multiple 1-2 mm sized T2 hyperintense renal cysts (black arrows) located within both the renal medulla and cortex.

**Fig. 4:**
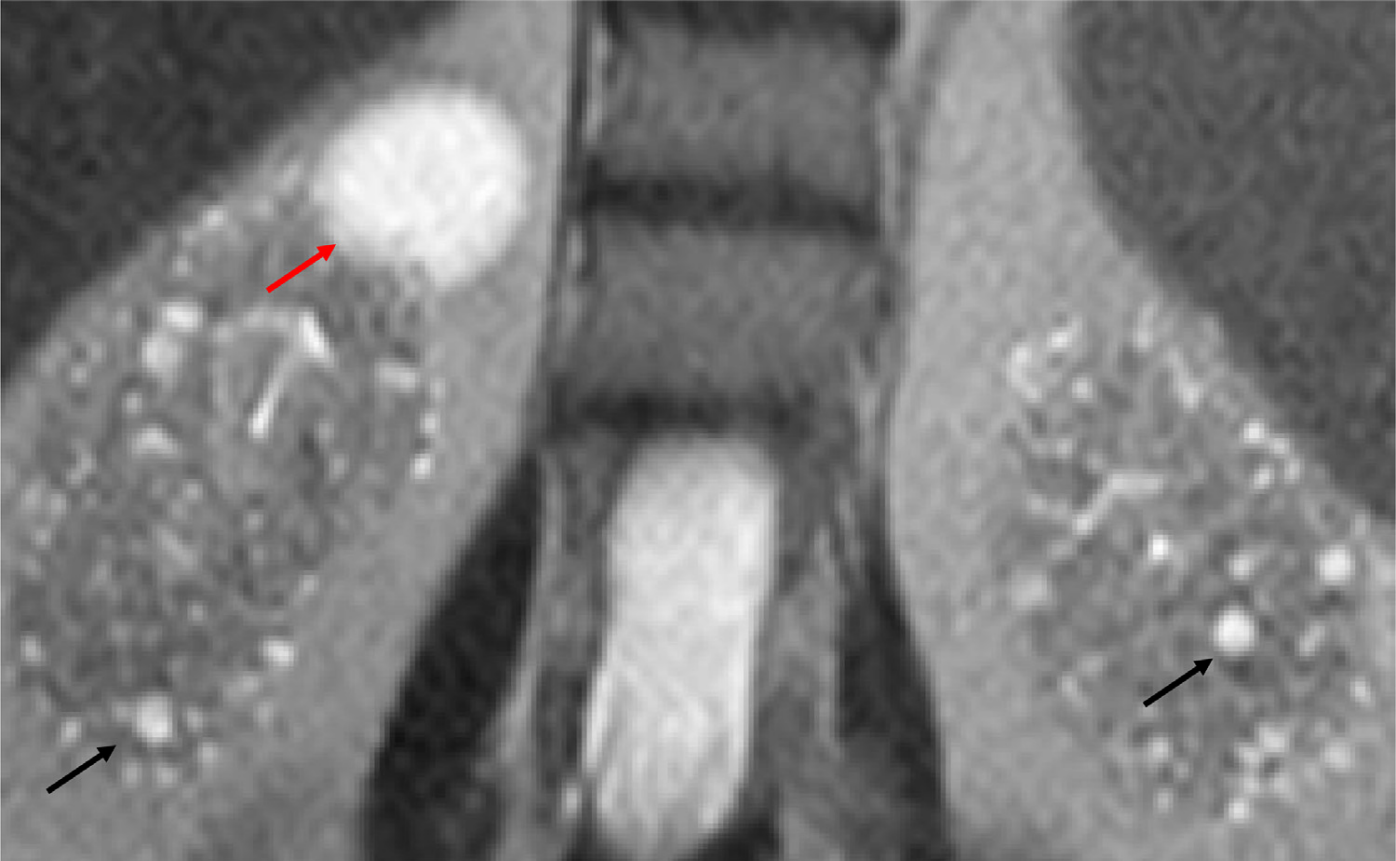
Select coronal T2 slice from MRI demonstrates normal-sized kidneys with multiple 1-2 mm sized T2 hyperintense renal cysts (black arrows) located within both the renal medulla and cortex. Additional, large simple renal cyst at the superior pole of the right kidney (red arrow).

**Fig. 5:**
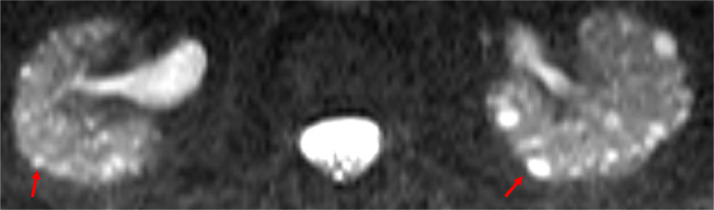
Select axial T2 slice from MRI demonstrates normal-sized kidneys with multiple 1-2 mm sized T2 hyperintense renal cysts (red arrows) located within both the renal medulla and cortex.

The point of care renal ultrasound performed in the office was concerning for unusual cystic change and scattered echogenic foci (Figs. 1 and 2). Further imaging characterization/evaluation was requested by nephrology with an MRI abdomen, renal mass protocol (Fig. 3, Fig. 4, Fig. 5). After further imaging evaluation, the patient was discussed at a multidisciplinary transplant clinic. It was determined that the patient's clinical picture and imaging findings were consistent with lithium-induced chronic renal disease. The patient did not meet the criteria (based on eGFR) for initiation of dialysis or the threshold to be listed for transplant. She will continue to be evaluated and followed by nephrology and if her renal function continues to decline, dialysis and transplant will be considered.

## Discussion

Lithium nephrotoxicity can be subdivided into 3 categories: acute intoxication, nephrogenic diabetes insipidus and chronic renal disease. The more acute forms of lithium nephrotoxicity have long been established. However, more recently after a wide epidemiologic review, the development of chronic renal disease has been correlated to lithium treatment alone adjusting for confounding factors [9]. Lithium-induced chronic renal disease is characterized by a progressive decline in renal function, measured by glomerular filtration rate (GFR) and creatinine clearance after treatment with lithium therapy for mood disorders.

These nephrotoxic effects occur due to the accumulation of lithium salts within the collecting renal tubular cells after entering via sodium ion channels. Within the tubular cells, lithium down-regulates vasopressin via G-coupled protein receptors, thus reducing the antidiuretic effect of vasopressin and may cause patients to become symptomatic including polydipsia and polyuria. This dysregulation can cause resistance to ADH and its ability to increase water permeability and provoke tubulointerstitial disease leading to the development of focal segmental glomerulosclerosis and microcyst formation. The risk of the development of renal disease after treatment with lithium has been shown to be age-dependent and increases with the duration of treatment with a hazard ratio of 2.5% (95% CI 1.6-4) [9]. These nephrotoxic effects of lithium are correlated to the length of treatment with an average latency between initiation of lithium therapy to end-stage renal disease of 20 years [10]. Persistent decline in renal function after cessation of lithium therapy may result. Although not performed in this case, renal biopsy results will demonstrate chronic tubulointerstitial nephropathy with associated cortical and medullary tubular cysts and dilation [9].

The unique imaging characteristics of lithium-induced renal disease aid in distinguishing this entity from other cystic renal pathology. On ultrasound, the findings of lithium renal disease can be suggested in the setting of multiple small cortical and medullary cysts with scattered echogenic calcifications. On magnetic resonance imaging, the findings of lithium renal disease can be identified by normal-sized kidneys with abundant medullary and cortically based T1 hypointense, T2 hyperintense, and well-circumscribed 1-2 mm cysts [8].

Differential considerations based on imaging findings include other cystic renal disease such as autosomal dominant polycystic kidney disease, medullary cystic disease, acquired cystic kidney disease and glomerulocystic kidney disease. Autosomal dominant polycystic kidney disease (ADPDK) is an inherited autosomal dominant cystic renal disease with PDK1 or PDK2 gene mutations. However, in patients with unknown genotype, the diagnosis can be made using the unified criteria via ultrasound stating the presence of three or more (unilateral or bilateral) cysts in individuals aged 15-39 years old, 2 or more cysts in each kidney in individuals aged 40-59 years old and 4 or more cysts in each kidney for individuals over the age of 60. In patients with ADPKD, the kidneys are enlarged rather than normal in size and the renal cysts vary in size and characteristic. Additionally, hepatic cysts can also be present in these patients [11].

Medullary cystic disease is an inherited cystic renal disease with associated renal dysfunction primarily seen in the pediatric population in which renal cysts of varying sizes are prominently medullary based in contrast to lithium renal disease where cysts are located both within the medulla and the cortex [11]. Additionally, the demographics of lithium chronic renal disease are predominately adults.

Acquired cystic kidney disease is an acquired condition that results from long-term chronic renal dysfunction and dialysis. The acquired renal cysts are located both within the medulla and cortex but vary in size. Additionally, patients are all undergoing dialysis [11].

Glomerulocystic kidney disease is a rare cystic renal disease characterized by dilation of the Bowman's capsule on pathology. The cysts are isolated to the renal cortex. On MR imaging, a T1 hypointense renal cortex with loss of corticomedullary differentiation and numerous cortical cysts is considered pathognomonic for glomerulocystic disease [12].

The treatment of lithium-induced chronic renal disease includes cessation of lithium therapy and transition to an alternative therapy if available [9]. The routine monitoring of renal function remains mandatory even after cessation of lithium therapy. A referral to nephrology for evaluation should be completed when appropriate [10].

## Conclusion

Despite the wide-spread use of lithium in mood disorders since the 1950s, the data confirming the correlation between lithium treatment and the development of chronic renal disease is relatively new secondary to recent large epidemiological reviews. In patients with a history of long-term lithium therapy and progressive renal dysfunction the unique imaging findings in lithium-induced renal disease provide an accurate, noninvasive means of clinical diagnosis.

## Patient consent

Complete written informed consent was obtained from the patient for the publication of this study and accompanying images.

## References

[1] Shorter E (2009). The history of lithium therapy. Bipolar Disord.

[2] Rowland TA, Marwaha S (2018). Epidemiology and risk factors for bipolar disorder. Ther Adv Psychopharmacol.

[3] Close H, Reilly J, Mason JM, Kripalani M, Wilson D, Main J (2014). Renal failure in lithium-treated bipolar disorder: a retrospective cohort study. PLoS One.

[4] Bocchetta A, Ardau R, Carta P, Ligas F, Sardu C, Pani A (2013). Duration of lithium treatment is a risk factor for reduced glomerular function: a cross-sectional study. BMC Med.

[5] McCann SM, Daly J, Kelly CB (2008). The impact of long-term lithium treatment on renal function in an outpatient population. Ulster Med J.

[6] Bendz H, Aurell M, Lanke J (2001). A historical cohort study of kidney damage in long-term lithium patients: continued surveillance needed. Eur Psychiatry.

[7] Kumarguru BN, Natrajan M, Nagarajappa AH (2013). The pathology of lithium induced nephropathy: a case report and review, with emphasis on the demonstration of mast cells. J Clin Diagn Res.

[8] Farres MT, Ronco P, Saadoun D, Remy P, Vincent F, Khalil A (2003). Chronic lithium nephropathy: MR imaging for diagnosis. Radiology.

[9] Azab AN, Shnaider A, Osher Y, Wang D, Bersudsky Y, Belmaker RH (2015). Lithium nephrotoxicity. Int J Bipolar Disord.

[10] Davis J, Desmond M, Berk M (2018). Lithium and nephrotoxicity: a literature review of approaches to clinical management and risk stratification. BMC Nephrol.

[11] Bisceglia M, Galliani CA, Senger C, Stallone C, Sessa A (2006). Renal cystic diseases: a review. Adv Anat Pathol.

[12] Oliva MR, Hsing J, Rybicki FJ, Fennessy F, Mortelé KJ, Ros PR (2003). Glomerulocystic kidney disease: MRI findings. Abdom Imaging.

